# Supplementary material for: Shelf Life of Minced Pork in Vacuum-Adsorbed Carvacrol@Natural Zeolite Nanohybrids and Poly-Lactic Acid/Triethyl Citrate/Carvacrol@Natural Zeolite Self-Healable Active Packaging Films
Source: Antioxidants (Basel). 2024 Jun 27;13(7):776. doi: 10.3390/antiox13070776 (PMC11274301; doi:10.3390/antiox13070776)
Supplement: Supplementary file 1 [file antioxidants-13-00776-s001.zip › antioxidants-3055095-supplementary.pdf]

Supplementary material for:

Shelf Life of Minced Pork in Vacuum-Adsorbed Carvacrol@Natural Zeolite Nanohybrids and Poly-Lactic Acid/Triethyl Citrate/Carvacrol@Natural Zeolite Self-Healable Active Packaging Films

Vassilios K. Karabagias [1], Aris E. Giannakas [1,*], Nikolaos D. Andritsos [1], Areti A. Leontiou [1], Dimitrios Moschovas [2], Andreas Karydis-Messinis [2], Apostolos Avgeropoulos [2], Nikolaos E. Zafeiropoulos [2], Charalampos Proestos [3] and Constantinos E. Salmas [2,*]

[1] Department of Food Science and Technology, University of Patras, 30100 Agrinio, Greece; vkarampagias@upatras.gr (V.K.K.); nandritsos@upatras.gr (N.D.A.) aleontiu@upatras.gr (A.A.L.)
[2] Department of Material Science and Engineering, University of Ioannina, 45110 Ioannina, Greece; dmoschov@uoi.gr (D.M.); karydis.and@gmail.com (A.K.-M.); aavger@uoi.gr (A.A.); nzafirop@uoi.gr (N.E.Z.)
[3] Laboratory of Food Chemistry, Department of Chemistry, National and Kapodistrian University of Athens Zografou, 15771 Athens, Greece; harpro@chem.uoa.gr
* Correspondence: agiannakas@upatras.gr (A.E.G.); ksalmas@uoi.gr (C.E.S.)

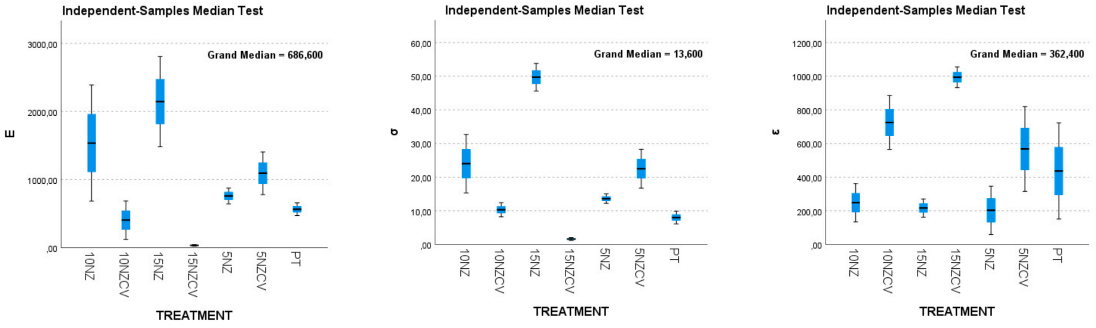

Figure S1: Independent-Samples Median Test for E, σ, %ε.

Table S1 : Pairwise Comparisons of the different treatments according to the mean values of E, σ, %ε.

E				σ				%ε			
**Pairwise Comparisons of TREATMENTS**				**Pairwise Comparisons of TREATMENTS**				**Pairwise Comparisons of TREATMENTS**			
Sample 1-Sample 2	Test Statistic	Sig.	Adj. Sig.[a]	Sample 1-Sample 2	Test Statistic	Sig.	Adj. Sig.[a]	Sample 1-Sample 2	Test Statistic	Sig.	Adj. Sig.[a]
15NZCV-10NZCV	6,000	0,014	0,300	15NZCV-PT	6,000	0,014	0,300	5NZ-15NZ	0,667	0,414	1,000
15NZCV-PT	6,000	0,014	0,300	15NZCV-10NZCV	6,000	0,014	0,300	5NZ-10NZ	0,667	0,414	1,000
15NZCV-5NZ	6,000	0,014	0,300	15NZCV-5NZ	6,000	0,014	0,300	5NZ-PT	0,667	0,414	1,000
15NZCV-5NZCV	6,000	0,014	0,300	15NZCV-5NZCV	6,000	0,014	0,300	5NZ-5NZCV	0,667	0,414	1,000
15NZCV-10NZ	6,000	0,014	0,300	15NZCV-10NZ	6,000	0,014	0,300	5NZ-10NZCV	6,000	0,014	0,300
15NZCV-15NZ	6,000	0,014	0,300	15NZCV-15NZ	6,000	0,014	0,300	5NZ-15NZCV	6,000	0,014	0,300
10NZCV-PT	0,667	0,414	1,000	PT-10NZCV	0,667	0,414	1,000	15NZ-10NZ	0,667	0,414	1,000
10NZCV-5NZ	0,667	0,414	1,000	PT-5NZ	6,000	0,014	0,300	15NZ-PT	0,667	0,414	1,000
10NZCV-5NZCV	6,000	0,014	0,300	PT-5NZCV	6,000	0,014	0,300	15NZ-5NZCV	6,000	0,014	0,300
10NZCV-10NZ	0,667	0,414	1,000	PT-10NZ	6,000	0,014	0,300	15NZ-10NZCV	6,000	0,014	0,300
10NZCV-15NZ	6,000	0,014	0,300	PT-15NZ	6,000	0,014	0,300	15NZ-15NZCV	6,000	0,014	0,300
PT-5NZ	0,667	0,414	1,000	10NZCV-5NZ	0,667	0,414	1,000	10NZ-PT	0,667	0,414	1,000
PT-5NZCV	6,000	0,014	0,300	10NZCV-5NZCV	6,000	0,014	0,300	10NZ-5NZCV	0,667	0,414	1,000
PT-10NZ	6,000	0,014	0,300	10NZCV-10NZ	6,000	0,014	0,300	10NZ-10NZCV	6,000	0,014	0,300
PT-15NZ	6,000	0,014	0,300	10NZCV-15NZ	6,000	0,014	0,300	10NZ-15NZCV	6,000	0,014	0,300
5NZ-5NZCV	0,667	0,414	1,000	5NZ-5NZCV	6,000	0,014	0,300	PT-5NZCV	0,667	0,414	1,000
5NZ-10NZ	0,667	0,414	1,000	5NZ-10NZ	6,000	0,014	0,300	PT-10NZCV	0,667	0,414	1,000
5NZ-15NZ	6,000	0,014	0,300	5NZ-15NZ	6,000	0,014	0,300	PT-15NZCV	6,000	0,014	0,300
5NZCV-10NZ	0,667	0,414	1,000	5NZCV-10NZ	0,667	0,414	1,000	5NZCV-10NZCV	0,667	0,414	1,000
5NZCV-15NZ	6,000	0,014	0,300	5NZCV-15NZ	6,000	0,014	0,300	5NZCV-15NZCV	6,000	0,014	0,300

10NZ-15NZ	0,667	0,414	1,000	10NZ-15NZ	6,000	0,014	0,300	10NZCV-15NZCV	6,000	0,014	0,300
Each row tests the null hypothesis that the Sample 1 and Sample 2 distributions are the same. Asymptotic significances (2-sided tests) are displayed. The significance level is ,050.				Each row tests the null hypothesis that the Sample 1 and Sample 2 distributions are the same. Asymptotic significances (2-sided tests) are displayed. The significance level is ,050.				Each row tests the null hypothesis that the Sample 1 and Sample 2 distributions are the same. Asymptotic significances (2-sided tests) are displayed. The significance level is ,050.			

Table S2: WVTR, D_{wv}, O.T.R. and Pe_{O2} values for all PLA/TEC/xNZ and PLA/TEC/xCV@NZ films as well

	**Film thickness (mm)**	**WVTR (10^{-7} gr.cm^{-2}. s^{1})**	**D_{wv} (10^{-4} cm^2/s)**	**Film thickness (mm)**	**OTR (ml.m^{-2}.day^{-1})**	**Pe_{O2} (10^{-9}cm^2/s)**
PLA/TEC	0.140±0.026	18.8573±6.65	3.69 ± 0.17^{a}	0.08±0.01	284.6±56.2	2.63 ± 0.55^{a}
PLA/TEC/5NZ	0.063±0.025	8.94097±0.73	2.76 ± 0.07^{b}	0.08±0.02	271.0±76.2	2.51 ± 0.05^{a}
PLA/TEC/10NZ	0.096±0.015	8.65331±0.34	2.26 ± 0.02^{c}	0.09±0.02	159.7±66.2	1.56 ± 0.07^{b}
PLA/TEC/15NZ	0.090±0.010	6.20586±0.29	1.72 ± 0.31^{d}	0.08±0.01	164.7±54.3	$1.48\pm0.65^{a,b}$
PLA/TEC/5CV@NZ	0.116±0.005	6.48645±0.15	$1.24\pm0.23^{d,e}$	0.12±0.01	105.4±15.7	1.52 ± 0.22^{b}
PLA/TEC/10CV@NZ	0.060±0.020	7.65358±0.20	$2.02\pm0.21^{f,d}$	0.07±0.01	187.3±36.4	1.59 ± 0.12^{b}
PLA/TEC/15CV@NZ	0.096±0.020	9.34063±0.30	2.35 ± 0.04^{g}	0.08±0.02	388.5±68.6	3.21 ± 0.23^{a}

as for pure PLA/TEC films.

Different letters in each column indicate statistically significant differences at the confidence level $p < 0.05$.

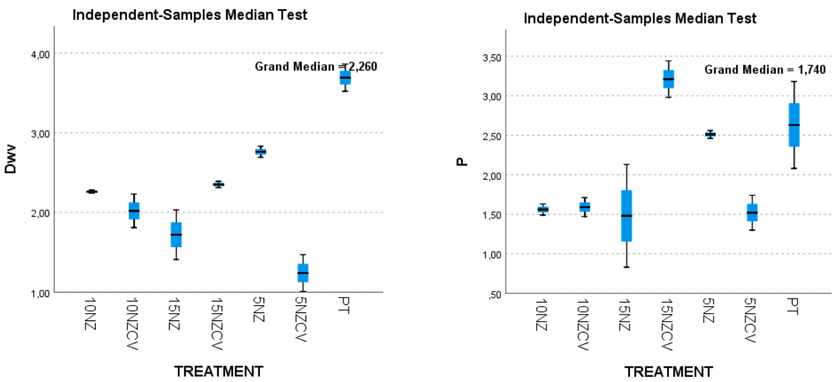

Figure S2 : Independent-Samples Median Test for Dwv,Peo2.

Table S3 : Pairwise Comparisons of the different treatments according to the mean values of Dwv,Peo2.

Dwv				Peo2			
**Pairwise Comparisons of TREATMENTS**				**Pairwise Comparisons of TREATMENTS**			
Sample 1-Sample 2	Test Statistic	Sig.	Adj. Sig.[a]	Sample 1-Sample 2	Test Statistic	Sig.	Adj. Sig.[a]
5NZCV-15NZ	0,667	0,414	1,000	15NZ-5NZCV	0,667	0,414	1,000
5NZCV-10NZCV	6,000	0,014	0,300	15NZ-10NZ	0,667	0,414	1,000

5NZCV-10NZ	6,000	0,014	0,300	15NZ-10NZCV	0,667	0,414	1,000
5NZCV-15NZCV	6,000	0,014	0,300	15NZ-5NZ	6,000	0,014	0,300
5NZCV-5NZ	6,000	0,014	0,300	15NZ-PT	0,667	0,414	1,000
5NZCV-PT	6,000	0,014	0,300	15NZ-15NZCV	6,000	0,014	0,300
15NZ-10NZCV	0,667	0,414	1,000	5NZCV-10NZ	0,667	0,414	1,000
15NZ-10NZ	6,000	0,014	0,300	5NZCV-10NZCV	0,667	0,414	1,000
15NZ-15NZCV	6,000	0,014	0,300	5NZCV-5NZ	6,000	0,014	0,300
15NZ-5NZ	6,000	0,014	0,300	5NZCV-PT	6,000	0,014	0,300
15NZ-PT	6,000	0,014	0,300	5NZCV-15NZCV	6,000	0,014	0,300
10NZCV-10NZ	6,000	0,014	0,300	10NZ-10NZCV	0,667	0,414	1,000
10NZCV-15NZCV	6,000	0,014	0,300	10NZ-5NZ	6,000	0,014	0,300
10NZCV-5NZ	6,000	0,014	0,300	10NZ-PT	6,000	0,014	0,300
10NZCV-PT	6,000	0,014	0,300	10NZ-15NZCV	6,000	0,014	0,300
10NZ-15NZCV	6,000	0,014	0,300	10NZCV-5NZ	6,000	0,014	0,300
10NZ-5NZ	6,000	0,014	0,300	10NZCV-PT	6,000	0,014	0,300
10NZ-PT	6,000	0,014	0,300	10NZCV-15NZCV	6,000	0,014	0,300
15NZCV-5NZ	6,000	0,014	0,300	5NZ-PT	0,667	0,414	1,000
15NZCV-PT	6,000	0,014	0,300	5NZ-15NZCV	6,000	0,014	0,300
5NZ-PT	6,000	0,014	0,300	PT-15NZCV	0,667	0,414	1,000
Each row tests the null hypothesis that the Sample 1 and Sample 2 distributions are the same. Asymptotic significances (2-sided tests) are displayed. The significance level is ,050.				Each row tests the null hypothesis that the Sample 1 and Sample 2 distributions are the same. Asymptotic significances (2-sided tests) are displayed. The significance level is ,050.			

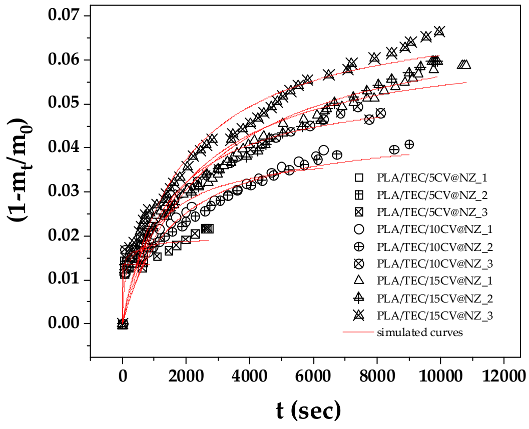

Figure S3: Plots of (1-mt/m0) as a function of time for all PLA/TEC/xCV@NZ samples (in triplicate) simulated with the pseudo second order kinetic equation to obtain k_2, and q_e mean values.

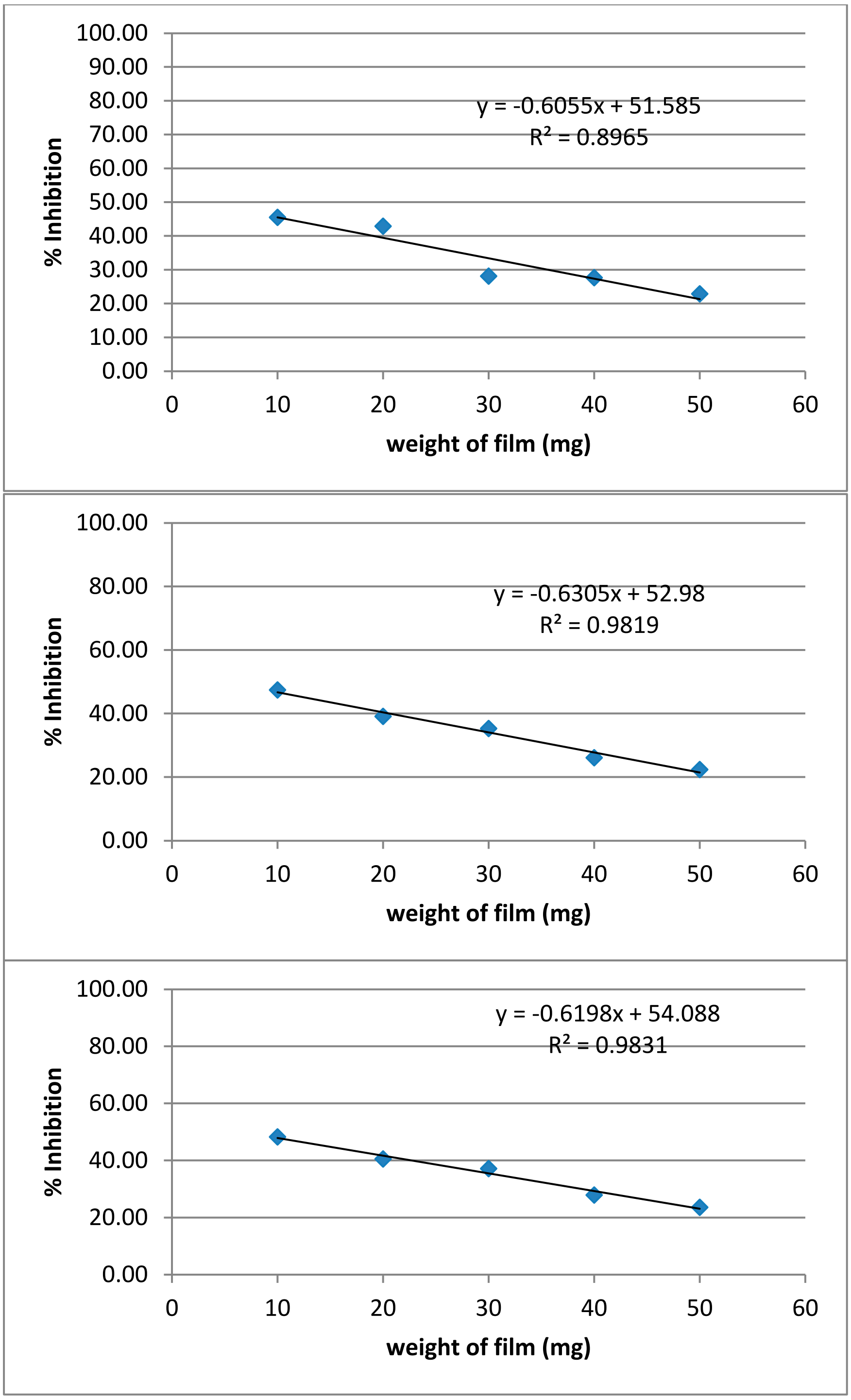

Figure S4: Equations for the determination of EC_{50} in PLA/TEC/5CV@NZ films.

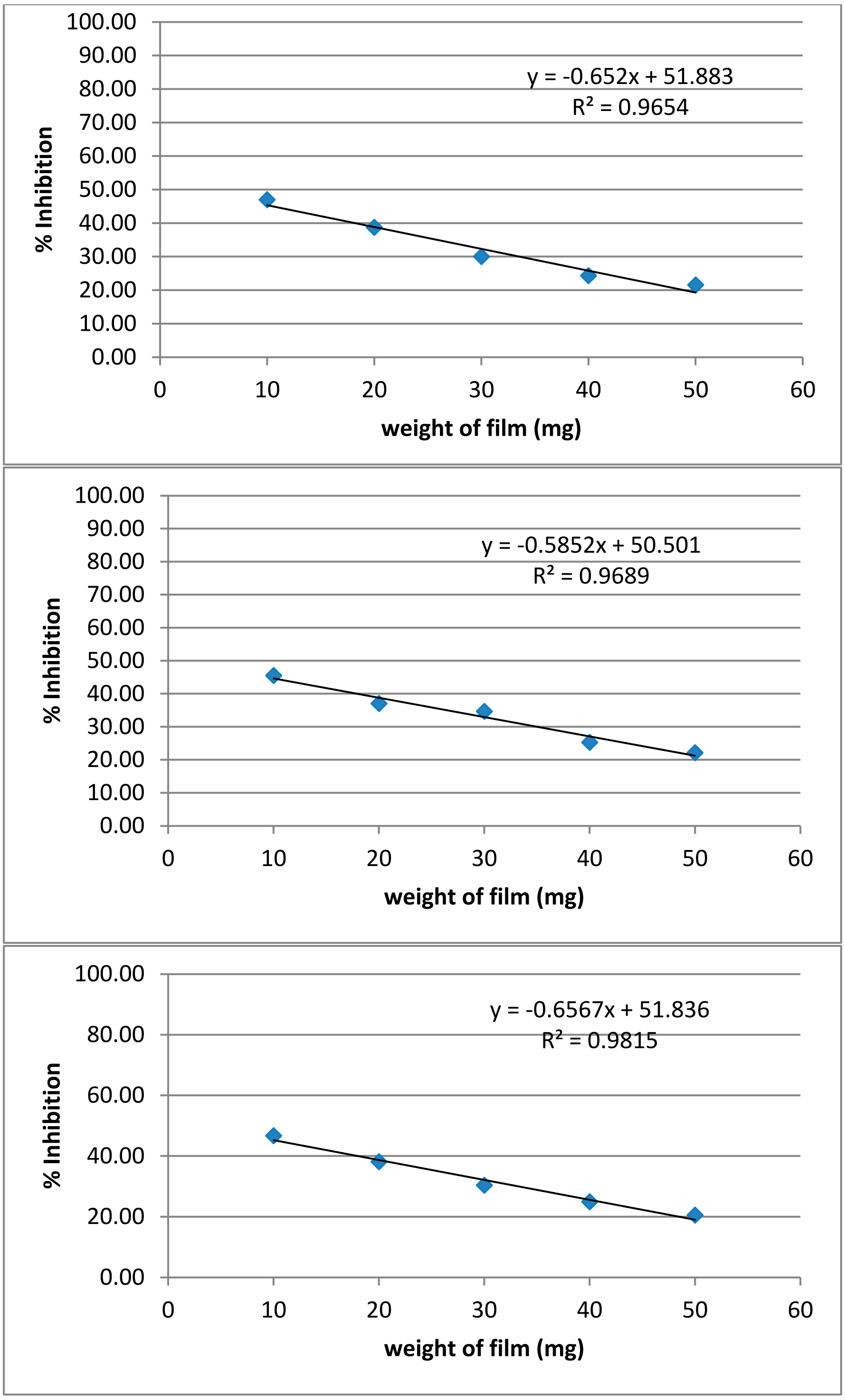

Figure S5: Equations for the determination of EC_{50} in PLA/TEC/10CV@NZ films.

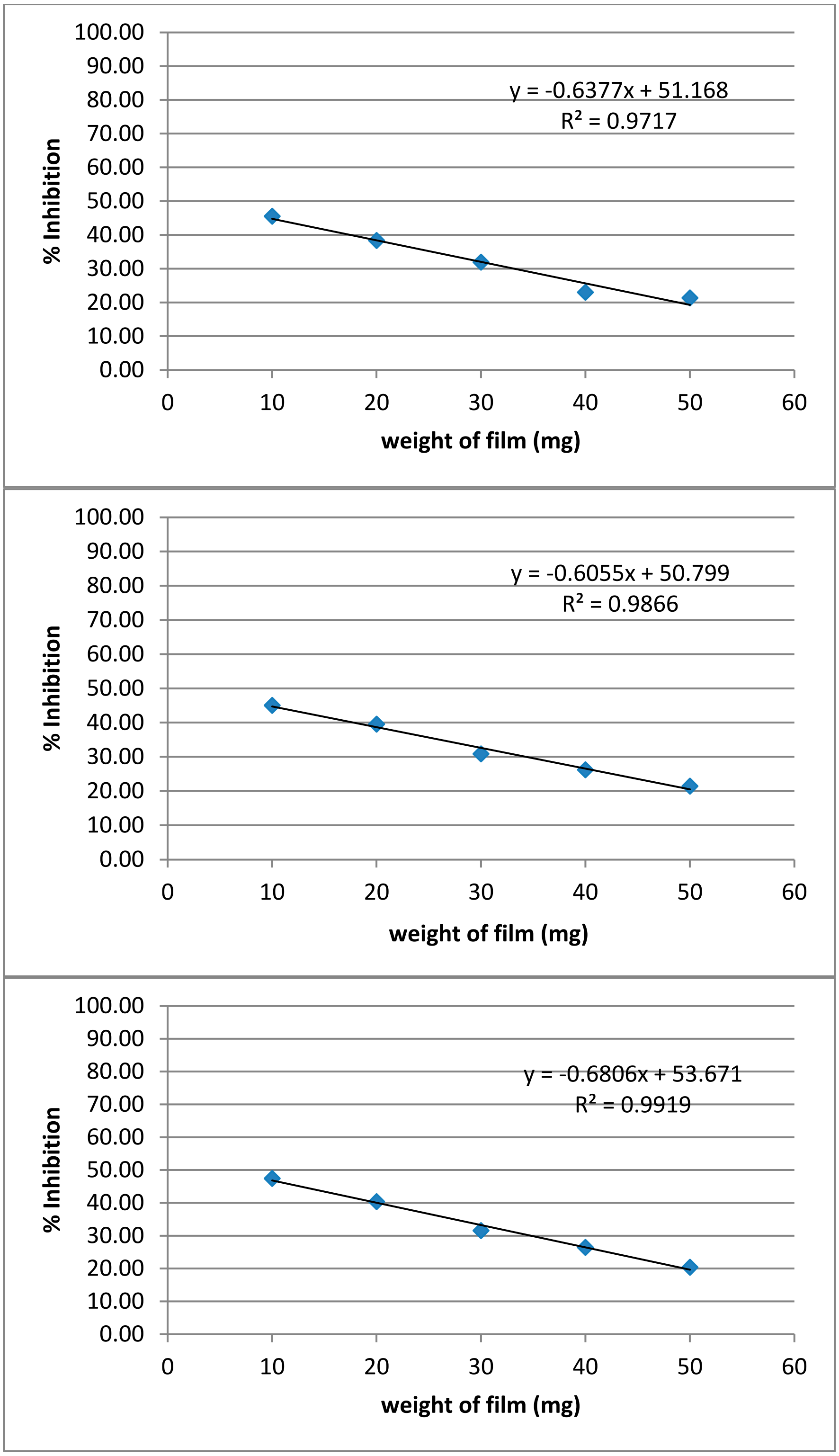

Figure S6: Equations for the determination of EC_{50} in PLA/TEC/15CV@NZ films.

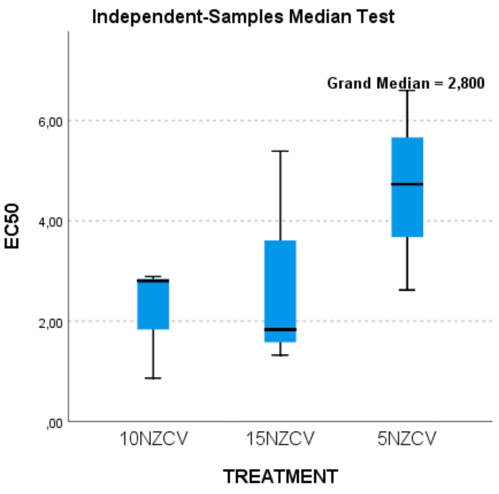

Figure S7 : Independent-Samples Median Test of EC_{50} values.

Table S4 : Independent-Samples Median Test Summary of the different treatments according to the mean values of EC_{50}.

Independent-Samples Median Test Summary

Total N	9
Median	2,800
Test Statistic	,900^{a,b}
Degree Of Freedom	2
Asymptotic Sig.(2-sided test)	,638

a. More than 20% of the cells have expected values less than five.

b. Multiple comparisons are not performed because the overall test does not show significant differences across samples.

Table S5: TVC of Pork Minced Meat in Different Packaging Systems with Respect to Storage Time.

Sample name	Day 0	Day 2	Day 4	Day 6	Day 8	Day 10
			logCFU/g (Avg ± SD)			
**CONTROL**	3.79±0.03^{a}	5.56±0.13^{b}	6.84±0.06^{e}	7.28±0.19^{h}	8.53±0.07^{k}	9.33±0.08^{n}
**PLA/TEC**	3.79±0.03^{a}	4.84±0.04^{c}	5.59±0.01^{f}	6.56±0.07^{i}	7.66±0.03^{l}	8.74±0.01^{o}
**PLA/TEC/10NZ@CV**	3.79±0.03^{a}	4.13±0.03^{d}	4.69±0.06^{g}	5.43±0.04^{j}	6.88±0.03^{m}	7.45±0.21^{p}

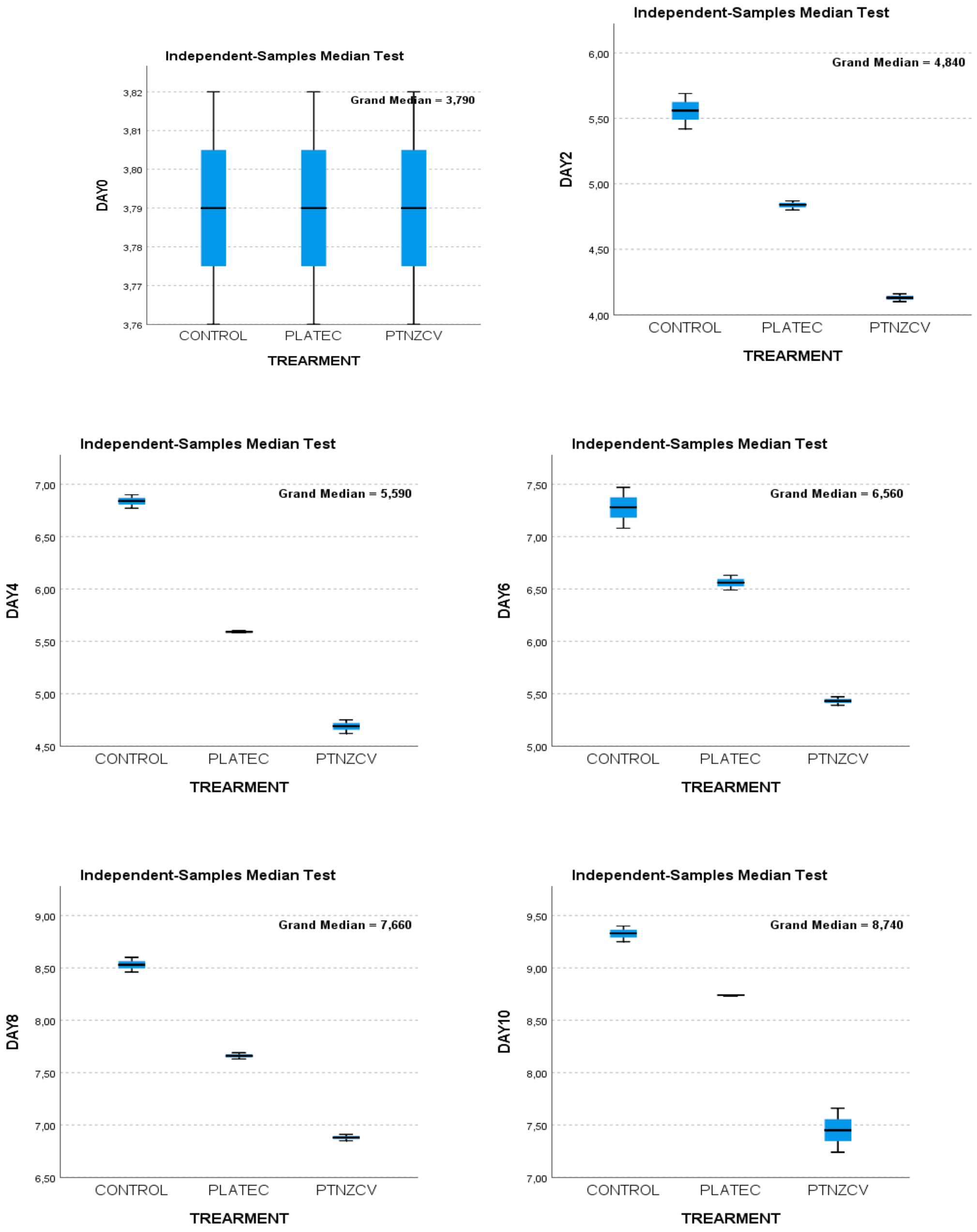

Figure S8: Independent-Samples Median Test of TVC durring storage time.

Table S6: Pairwise Comparisons of the different treatments according to the mean values of TVC during storage time.

DAY 2

Pairwise Comparisons of TREATMENTS

Sample 1-Sample 2	Test Statistic	Sig.	Adj. Sig.[a]
PTNZCV-PLATEC	6,000	0,014	0,043
PTNZCV-CONTROL	6,000	0,014	0,043
PLATEC-CONTROL	6,000	0,014	0,043

Each row tests the null hypothesis that the Sample 1 and Sample 2 distributions are the same.
Asymptotic significances (2-sided tests) are displayed. The significance level is ,05.

a. Significance values have been adjusted by the Bonferroni correction for multiple tests.

DAY4

Pairwise Comparisons of TREATMENTS

Sample 1-Sample 2	Test Statistic	Sig.	Adj. Sig.[a]
PTNZCV-PLATEC	6,000	0,014	0,043
PTNZCV-CONTROL	6,000	0,014	0,043
PLATEC-CONTROL	6,000	0,014	0,043

Each row tests the null hypothesis that the Sample 1 and Sample 2 distributions are the same.
Asymptotic significances (2-sided tests) are displayed. The significance level is ,05.

a. Significance values have been adjusted by the Bonferroni correction for multiple tests.

DAY6

Pairwise Comparisons of TREATMENTS

Sample 1-Sample 2	Test Statistic	Sig.	Adj. Sig.[a]
PTNZCV-PLATEC	6,000	0,014	0,043
PTNZCV-CONTROL	6,000	0,014	0,043
PLATEC-CONTROL	6,000	0,014	0,043

Each row tests the null hypothesis that the Sample 1 and Sample 2 distributions are the same.
Asymptotic significances (2-sided tests) are displayed. The significance level is ,05.

a. Significance values have been adjusted by the Bonferroni correction for multiple tests.

DAY8

Pairwise Comparisons of TREATMENTS

Sample 1-Sample 2	Test Statistic	Sig.	Adj. Sig.[a]
PTNZCV-PLATEC	6,000	0,014	0,043
PTNZCV-CONTROL	6,000	0,014	0,043
PLATEC-CONTROL	6,000	0,014	0,043

Each row tests the null hypothesis that the Sample 1 and Sample 2 distributions are the same.
Asymptotic significances (2-sided tests) are displayed. The significance level is ,05.

a. Significance values have been adjusted by the Bonferroni correction for multiple tests.

DAY10

Pairwise Comparisons of TREATMENTS

Sample 1-Sample 2	Test Statistic	Sig.	Adj. Sig.[a]
PTNZCV-PLATEC	6,000	0,014	0,043
PTNZCV-CONTROL	6,000	0,014	0,043
PLATEC-CONTROL	6,000	0,014	0,043

Each row tests the null hypothesis that the Sample 1 and Sample 2 distributions are the same.
Asymptotic significances (2-sided tests) are displayed. The significance level is ,05.

a. Significance values have been adjusted by the Bonferroni correction for multiple tests.

Table S7: TBARS and Heme-Iron Content of Pork Minced Meat in Different Packaging Systems with Respect to Storage Time.

TBARS	*Day 0*	*Day 2*	*Day 4*	*Day 6*	*Day 8*	*Day 10*
			**AVG ± SD (mg/kg)**			
Control	0.29 ± 0.02^{a}	0.43 ± 0.01^{b}	0.55± 0.02^{e}	0.65 ± 0.02^{h}	0.77 ± 0.03^{k}	0.93 ± 0.01^{m}
PLA/TEC	-	0.40 ± 0.01^{c}	0.51 ± 0.01^{f}	0.60± 0.02^{i}	0.73 ± 0.01^{k}	0.87 ± 0.01^{n}
PLA/TEC/0.6/NZCV	-	0.36 ± 0.01^{d}	0.43 ± 0.01^{g}	0.54 ± 0.01^{j}	0.67 ± 0.01^{l}	0.78± 0.01^{o}
**Fe**	*Day 0*	*Day 2*	*Day 4*	*Day 6*	*Day 8*	*Day 10*
			**AVG ± SD (μg/g)**			
Control	11.12 ± 0.15^{a}	9.30 ± 0.18^{b}	8.18 ± 0.12^{d}	6.76 ± 0.21^{g}	4.90± 0.18^{j}	3.48 ± 0.36^{m}
PLA/TEC	-	9.48 ± 0.22^{b}	8.56 ± 0.28^{e}	7.24 ± 0.23^{h}	5.64 ± 0.22^{k}	3.92 ± 0.27^{m}
PLA/TEC/0.6/NZCV	-	10.02 ± 0.22^{c}	9.30 ± 0.22^{f}	7.98 ± 0.27^{i}	7.18 ± 0.09^{l}	4.42 ± 0.12^{n}

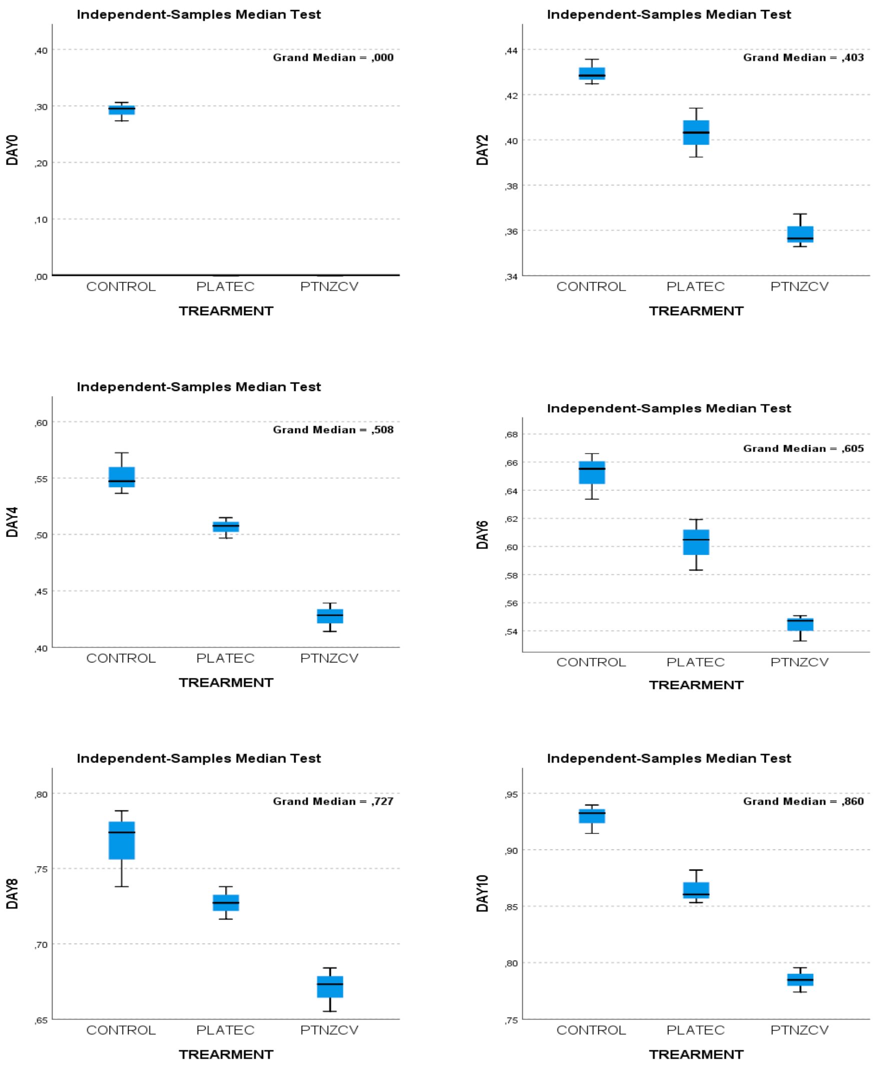

Figure S9: Independent-Samples Median Test of TBARS durring storage time.

Table S8: Pairwise Comparisons of the different treatments according to the mean values of TBARS during storage time.

DAY2

Pairwise Comparisons of TREATMENTS

Sample 1-Sample 2	Test Statistic	Sig.	Adj. Sig.[a]
PTNZCV-PLATEC	6,000	0,014	0,043
PTNZCV-CONTROL	6,000	0,014	0,043
PLATEC-CONTROL	6,000	0,014	0,043

Each row tests the null hypothesis that the Sample 1 and Sample 2 distributions are the same.
Asymptotic significances (2-sided tests) are displayed. The significance level is ,05.

a. Significance values have been adjusted by the Bonferroni correction for multiple tests.

DAY4

Pairwise Comparisons of TREATMENTS

Sample 1-Sample 2	Test Statistic	Sig.	Adj. Sig.[a]
PTNZCV-PLATEC	6,000	0,014	0,043
PTNZCV-CONTROL	6,000	0,014	0,043
PLATEC-CONTROL	6,000	0,014	0,043

Each row tests the null hypothesis that the Sample 1 and Sample 2 distributions are the same.
Asymptotic significances (2-sided tests) are displayed. The significance level is ,05.

a. Significance values have been adjusted by the Bonferroni correction for multiple tests.

DAY6

Pairwise Comparisons of TREATMENTS

Sample 1-Sample 2	Test Statistic	Sig.	Adj. Sig.[a]
PTNZCV-PLATEC	6,000	0,014	0,043
PTNZCV-CONTROL	6,000	0,014	0,043
PLATEC-CONTROL	6,000	0,014	0,043

Each row tests the null hypothesis that the Sample 1 and Sample 2 distributions are the same.
Asymptotic significances (2-sided tests) are displayed. The significance level is ,05.

a. Significance values have been adjusted by the Bonferroni correction for multiple tests.

DAY8

Pairwise Comparisons of TREATMENTS

Sample 1-Sample 2	Test Statistic	Sig.	Adj. Sig.[a]
PTNZCV-PLATEC	6,000	0,014	0,043
PTNZCV-CONTROL	6,000	0,014	0,043
PLATEC-CONTROL	3,000	0,083	0,250

Each row tests the null hypothesis that the Sample 1 and Sample 2 distributions are the same.
Asymptotic significances (2-sided tests) are displayed. The significance level is ,05.

a. Significance values have been adjusted by the Bonferroni correction for multiple tests.

DAY10

Pairwise Comparisons of TREATMENTS

Sample 1-Sample 2	Test Statistic	Sig.	Adj. Sig.[a]
PTNZCV-PLATEC	6,000	0,014	0,043
PTNZCV-CONTROL	6,000	0,014	0,043
PLATEC-CONTROL	6,000	0,014	0,043

Each row tests the null hypothesis that the Sample 1 and Sample 2 distributions are the same.
Asymptotic significances (2-sided tests) are displayed. The significance level is ,05.

a. Significance values have been adjusted by the Bonferroni correction for multiple tests.

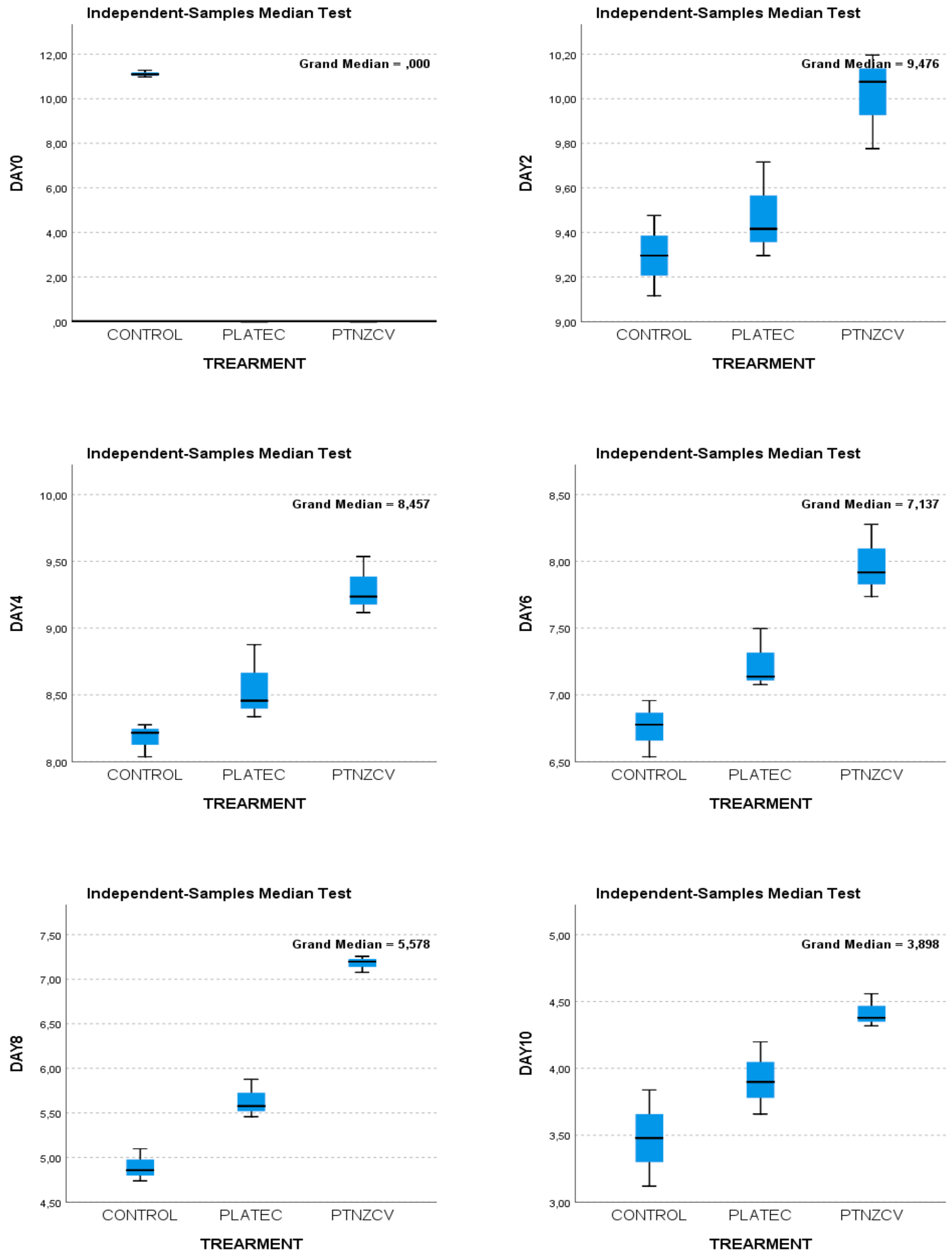

Figure S10 : Independent-Samples Median Test of Heme-iron content durring storage time.

Table S9: Pairwise Comparisons of the different treatments according to the mean values of Heme-iron content during storage time.

DAY2				DAY4				DAY6			
**Pairwise Comparisons of TREATMENTS**				**Pairwise Comparisons of TREATMENTS**				**Pairwise Comparisons of TREATMENTS**			
Sample 1-Sample 2	Test Statistic	Sig.	Adj. Sig.[a]	Sample 1-Sample 2	Test Statistic	Sig.	Adj. Sig.[a]	Sample 1-Sample 2	Test Statistic	Sig.	Adj. Sig.[a]
CONTROL-PLATEC	0,667	0,414	1,000	CONTROL-PLATEC	6,000	0,014	0,043	CONTROL-PLATEC	6,000	0,014	0,043
CONTROL-PTNZCV	6,000	0,014	0,043	CONTROL-PTNZCV	6,000	0,014	0,043	CONTROL-PTNZCV	6,000	0,014	0,043
PLATEC-PTNZCV	6,000	0,014	0,043	PLATEC-PTNZCV	6,000	0,014	0,043	PLATEC-PTNZCV	6,000	0,014	0,043
Each row tests the null hypothesis that the Sample 1 and Sample 2 distributions are the same. Asymptotic significances (2-sided tests) are displayed. The significance level is ,05.				Each row tests the null hypothesis that the Sample 1 and Sample 2 distributions are the same. Asymptotic significances (2-sided tests) are displayed. The significance level is ,05.				Each row tests the null hypothesis that the Sample 1 and Sample 2 distributions are the same. Asymptotic significances (2-sided tests) are displayed. The significance level is ,05.			
a. Significance values have been adjusted by the Bonferroni correction for multiple tests.				a. Significance values have been adjusted by the Bonferroni correction for multiple tests.				a. Significance values have been adjusted by the Bonferroni correction for multiple tests.			

DAY8				DAY10			
**Pairwise Comparisons of TREATMENTS**				**Pairwise Comparisons of TREATMENTS**			
Sample 1-Sample 2	Test Statistic	Sig.	Adj. Sig.[a]	Sample 1-Sample 2	Test Statistic	Sig.	Adj. Sig.[a]
CONTROL-PLATEC	6,000	0,014	0,043	CONTROL-PLATEC	0,667	0,414	1,000
CONTROL-PTNZCV	6,000	0,014	0,043	CONTROL-PTNZCV	6,000	0,014	0,043
PLATEC-PTNZCV	6,000	0,014	0,043	PLATEC-PTNZCV	6,000	0,014	0,043
Each row tests the null hypothesis that the Sample 1 and Sample 2 distributions are the same. Asymptotic significances (2-sided tests) are displayed. The significance level is ,05.				Each row tests the null hypothesis that the Sample 1 and Sample 2 distributions are the same. Asymptotic significances (2-sided tests) are displayed. The significance level is ,05.			
a. Significance values have been adjusted by the Bonferroni correction for multiple tests.				a. Significance values have been adjusted by the Bonferroni correction for multiple tests.			

Table S10 : Pearson's Correlation between TBARS and Heme-iron content during storage time.

Correlations

		DAY0	DAY2	DAY4	DAY6	DAY8	DAY10
DAY0	Pearson Correlation	1	,408	,381	,360	,255	,298
	Sig. (2-tailed)		,093	,118	,142	,307	,229
	N	18	18	18	18	18	18
DAY2	Pearson Correlation	,408	1	,999**	,997**	,978**	,988**
	Sig. (2-tailed)	,093		,000	,000	,000	,000
	N	18	18	18	18	18	18

DAY4	Pearson Correlation	,381	,999**	1	,998**	,984**	,992**
	Sig. (2-tailed)	,118	,000		,000	,000	,000
	N	18	18	18	18	18	18
DAY6	Pearson Correlation	,360	,997**	,998**	1	,988**	,991**
	Sig. (2-tailed)	,142	,000	,000		,000	,000
	N	18	18	18	18	18	18
DAY8	Pearson Correlation	,255	,978**	,984**	,988**	1	,993**
	Sig. (2-tailed)	,307	,000	,000	,000		,000
	N	18	18	18	18	18	18
DAY10	Pearson Correlation	,298	,988**	,992**	,991**	,993**	1
	Sig. (2-tailed)	,229	,000	,000	,000	,000	
	N	18	18	18	18	18	18

**. Correlation is significant at the 0.01 level (2-tailed).

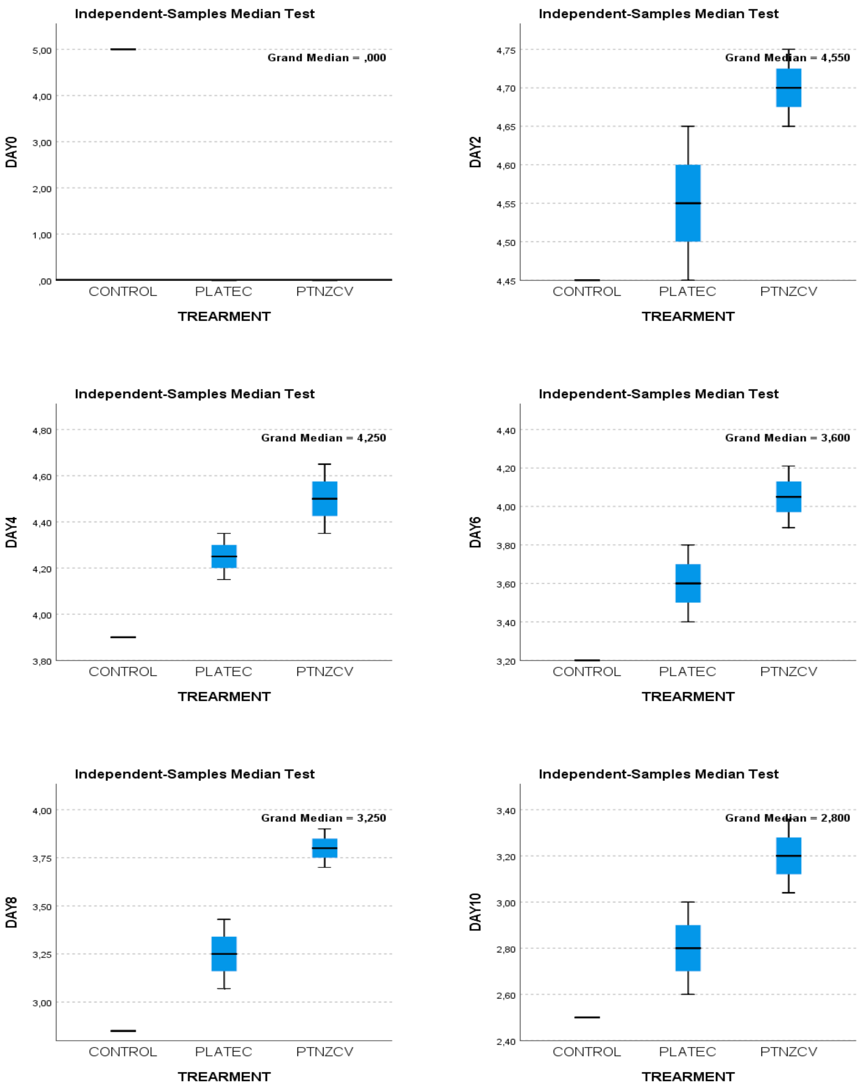

Figure S11 : Independent-Samples Median Test of Odor durring storage time.

Table S11: Pairwise Comparisons of the different treatments according to the mean values of Odor during storage time.

DAY2

Pairwise Comparisons of TREATMENTS

Sample 1-Sample 2	Test Statistic	Sig.	Adj. Sig.[a]
CONTROL-PLATEC	3,000	0,083	0,250
CONTROL-PTNZCV	6,000	0,014	0,043
PLATEC-PTNZCV	3,000	0,083	0,250

Each row tests the null hypothesis that the Sample 1 and Sample 2 distributions are the same.
Asymptotic significances (2-sided tests) are displayed. The significance level is ,05.

a. Significance values have been adjusted by the Bonferroni correction for multiple tests.

DAY4

Pairwise Comparisons of TREATMENTS

Sample 1-Sample 2	Test Statistic	Sig.	Adj. Sig.[a]
CONTROL-PLATEC	6,000	0,014	0,043
CONTROL-PTNZCV	6,000	0,014	0,043
PLATEC-PTNZCV	3,000	0,083	0,250

Each row tests the null hypothesis that the Sample 1 and Sample 2 distributions are the same.
Asymptotic significances (2-sided tests) are displayed. The significance level is ,05.

a. Significance values have been adjusted by the Bonferroni correction for multiple tests.

DAY6

Pairwise Comparisons of TREATMENTS

Sample 1-Sample 2	Test Statistic	Sig.	Adj. Sig.[a]
CONTROL-PLATEC	6,000	0,014	0,043
CONTROL-PTNZCV	6,000	0,014	0,043
PLATEC-PTNZCV	6,000	0,014	0,043

Each row tests the null hypothesis that the Sample 1 and Sample 2 distributions are the same.
Asymptotic significances (2-sided tests) are displayed. The significance level is ,05.

a. Significance values have been adjusted by the Bonferroni correction for multiple tests.

DAY8

Pairwise Comparisons of TREATMENTS

Sample 1-Sample 2	Test Statistic	Sig.	Adj. Sig.[a]
CONTROL-PLATEC	6,000	0,014	0,043
CONTROL-PTNZCV	6,000	0,014	0,043
PLATEC-PTNZCV	6,000	0,014	0,043

Each row tests the null hypothesis that the Sample 1 and Sample 2 distributions are the same.
Asymptotic significances (2-sided tests) are displayed. The significance level is ,05.

DAY10

Pairwise Comparisons of TREATMENTS

Sample 1-Sample 2	Test Statistic	Sig.	Adj. Sig.[a]
CONTROL-PLATEC	6,000	0,014	0,043
CONTROL-PTNZCV	6,000	0,014	0,043
PLATEC-PTNZCV	6,000	0,014	0,043

Each row tests the null hypothesis that the Sample 1 and Sample 2 distributions are the same.
Asymptotic significances (2-sided tests) are displayed. The significance level is ,05.

a. Significance values have been adjusted by the Bonferroni correction for multiple tests.	a. Significance values have been adjusted by the Bonferroni correction for multiple tests.

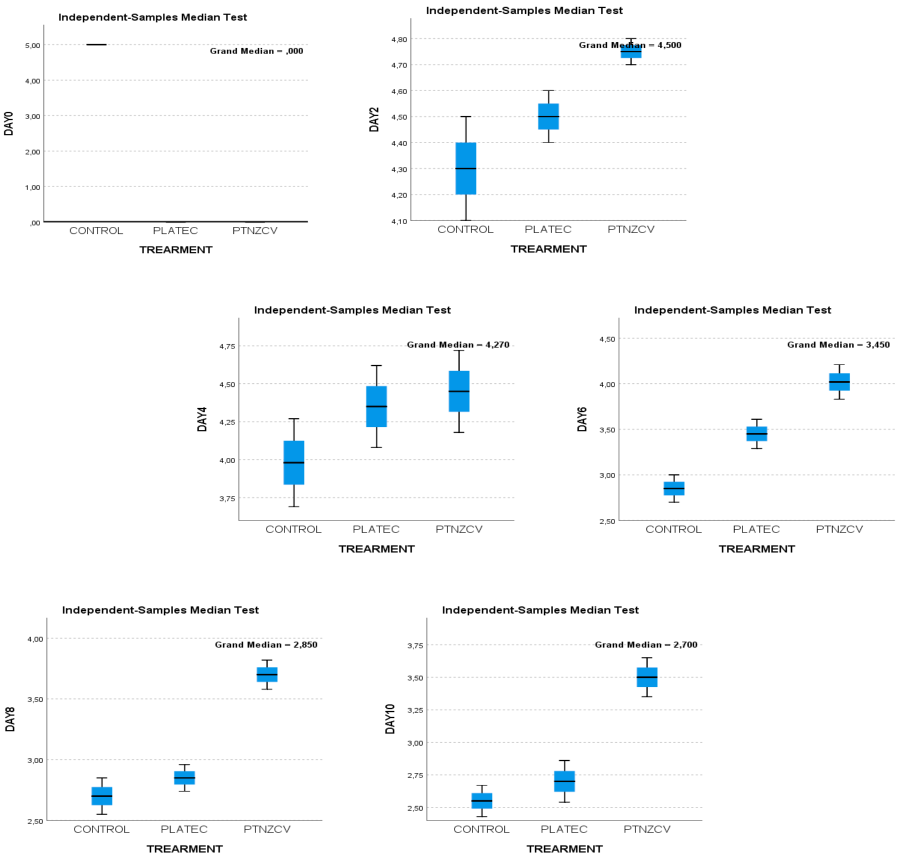

Figure S12: Independent-Samples Median Test of Color durring storage time.

Table S12: Pairwise Comparisons of the different treatments according to the mean values of Color during storage time.

DAY2 — Pairwise Comparisons of TREATMENTS

Sample 1-Sample 2	Test Statistic	Sig.	Adj. Sig.[a]
CONTROL-PLATEC	0,667	0,414	1,000
CONTROL-PTNZCV	6,000	0,014	0,043
PLATEC-PTNZCV	6,000	0,014	0,043

Each row tests the null hypothesis that the Sample 1 and Sample 2 distributions are the same.
Asymptotic significances (2-sided tests) are displayed. The significance level is ,05.

a. Significance values have been adjusted by the Bonferroni correction for multiple tests.

DAY4 — Independent-Samples Median Test Summary

Total N	9
Median	4,270
Test Statistic	3,600[a,b]
Degree Of Freedom	2
Asymptotic Sig.(2-sided test)	

a. More than 20% of the cells have expected values less than five.

b. Multiple comparisons are not performed because the overall test does not show significant differences across samples.

DAY6 — Pairwise Comparisons of TREATMENTS

Sample 1-Sample 2	Test Statistic	Sig.	Adj. Sig.[a]
CONTROL-PLATEC	6,000	0,014	0,043
CONTROL-PTNZCV	6,000	0,014	0,043
PLATEC-PTNZCV	6,000	0,014	0,043

Each row tests the null hypothesis that the Sample 1 and Sample 2 distributions are the same.
Asymptotic significances (2-sided tests) are displayed. The significance level is ,05.

a. Significance values have been adjusted by the Bonferroni correction for multiple tests.

DAY8 — Pairwise Comparisons of TREATMENTS

Sample 1-Sample 2	Test Statistic	Sig.	Adj. Sig.[a]
CONTROL-PLATEC	0,667	0,414	1,000
CONTROL-PTNZCV	6,000	0,014	0,043
PLATEC-PTNZCV	6,000	0,014	0,043

Each row tests the null hypothesis that the Sample 1 and Sample 2 distributions are the same.
Asymptotic significances (2-sided tests) are displayed. The significance level is ,05.

a. Significance values have been adjusted by the Bonferroni correction for multiple tests.

DAY10 — Pairwise Comparisons of TREATMENTS

Sample 1-Sample 2	Test Statistic	Sig.	Adj. Sig.[a]
CONTROL-PLATEC	0,667	0,414	1,000
CONTROL-PTNZCV	6,000	0,014	0,043
PLATEC-PTNZCV	6,000	0,014	0,043

Each row tests the null hypothesis that the Sample 1 and Sample 2 distributions are the same.
Asymptotic significances (2-sided tests) are displayed. The significance level is ,05.

a. Significance values have been adjusted by the Bonferroni correction for multiple tests.

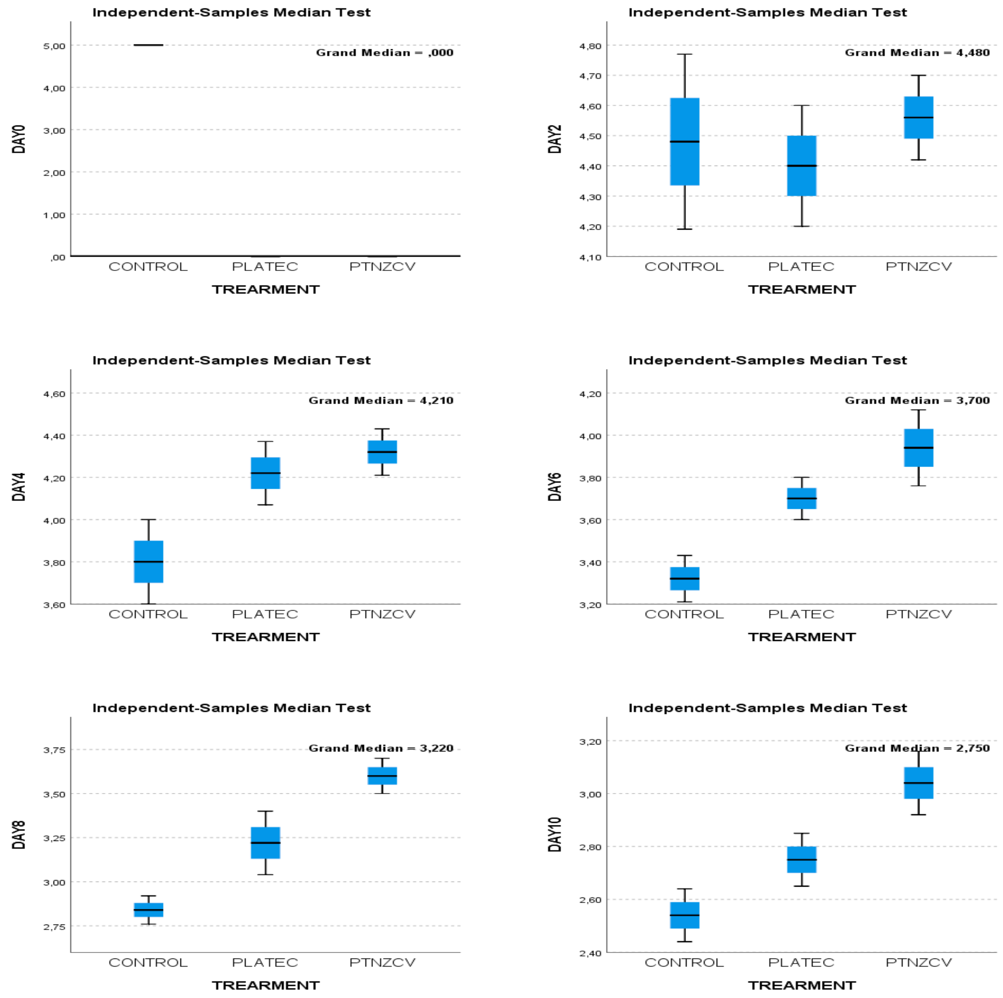

Figure S13: Independent-Samples Median Test of Texture durring storage time.

Table S13: Pairwise Comparisons of the different treatments according to the mean values of Texture during storage time.

DAY2		DAY4	
**Independent-Samples Median Test Summary**		**Independent-Samples Median Test Summary**	
Total N	9	Total N	9
Median	4,480	Median	4,210
Test Statistic	,900[a,b]	Test Statistic	3,600[a,b]
Degree Of Freedom	2	Degree Of Freedom	2
Asymptotic Sig.(2-sided test)	0,638	Asymptotic Sig.(2-sided test)	0,165
a. More than 20% of the cells have expected values less than five.		a. More than 20% of the cells have expected values less than five.	
b. Multiple comparisons are not performed because the overall test does not show significant differences across samples.		b. Multiple comparisons are not performed because the overall test does not show significant differences across samples.	

DAY6			
**Pairwise Comparisons of TREATMENTS**			
Sample 1-Sample 2	Test Statistic	Sig.	Adj. Sig.[a]
CONTROL-PLATEC	6,000	0,014	0,043
CONTROL-PTNZCV	6,000	0,014	0,043
PLATEC-PTNZCV	0,667	0,414	1,000
Each row tests the null hypothesis that the Sample 1 and Sample 2 distributions are the same. Asymptotic significances (2-sided tests) are displayed. The significance level is ,05.			
a. Significance values have been adjusted by the Bonferroni correction for multiple tests.			

DAY8			
**Pairwise Comparisons of TREATMENTS**			
Sample 1-Sample 2	Test Statistic	Sig.	Adj. Sig.[a]
CONTROL-PLATEC	6,000	0,014	0,043
CONTROL-PTNZCV	6,000	0,014	0,043
PLATEC-PTNZCV	6,000	0,014	0,043
Each row tests the null hypothesis that the Sample 1 and Sample 2 distributions are the same. Asymptotic significances (2-sided tests) are displayed. The significance level is ,05.			
a. Significance values have been adjusted by the Bonferroni correction for multiple tests.			

DAY10			
**Pairwise Comparisons of TREATMENTS**			
Sample 1-Sample 2	Test Statistic	Sig.	Adj. Sig.[a]
CONTROL-PLATEC	6,000	0,014	0,043
CONTROL-PTNZCV	6,000	0,014	0,043
PLATEC-PTNZCV	6,000	0,014	0,043
Each row tests the null hypothesis that the Sample 1 and Sample 2 distributions are the same. Asymptotic significances (2-sided tests) are displayed. The significance level is ,05.			
a. Significance values have been adjusted by the Bonferroni correction for multiple tests.			
